# Assessing Environmentally Effective Post-COVID Green Recovery Plans for Reducing Social and Economic Inequality

**DOI:** 10.1007/s44177-022-00037-x

**Published:** 2022-09-16

**Authors:** James R. Sokolnicki, Annabel L. Woodhatch, Richard Stafford

**Affiliations:** Department of Life and Environmental Sciences, Bournemouth University, Fern Barrow, Poole, BH12 5BB UK

**Keywords:** Green New Deal, Nature-based solutions, Green recovery, Environmental breakdown, Bayesian belief network

## Abstract

Given the current environmental crisis there have been multiple calls for a green recovery from COVID-19 which address environmental concerns and provide jobs in industries and communities economically damaged by the pandemic. Here, we holistically evaluate a range of recovery scenarios, evaluated on environmental and socio-economic equity metrics. Using a modified version of a Bayesian belief network, we show that economic stimuli across green sectors, including jobs in renewable energy, waste management, retrofitting of buildings, heat-pump installation and public transport can help economic growth, but will have limited environmental benefits. The inclusion of carbon taxes and ending fossil fuel subsidies, alongside investment in nature-based solutions and jobs in ecological conservation, can greatly increase the environmental gains as well as socio-economic equality. Additionally, jobs not associated with green industries, but with low carbon footprints, such as those in social care can further improve social equality with minimal negative environmental effects. However, in these latter scenarios involving taxation and ending fossil fuel subsidies, economic growth is reduced. We suggest a comprehensive green recovery and green new deal are needed, and we should reimagine economies, without the focus on economic growth.

**Supplementary Information:**

The online version contains supplementary material available at 10.1007/s44177-022-00037-x.

## Introduction

COVID-19 brought havoc to a vulnerable world—already damaged by biodiversity loss, climate change, over-exploitation of resources, globalisation, social inequalities, unsustainable consumption and production, and inadequately prepared governments (Stafford and Jones 2019). Currently, the world is at a critical junction, and COVID-19 presents a window of opportunity that is rapidly closing to guide future development within the Earth’s planetary boundaries, and in an operating space safe for humans (Steffen et al. 2015; Helm 2020; Lenzen et al. 2020; Frutos et al. 2021). Governments have been urged by individuals, NGOs and think-tanks to pursue a resilient and ‘Green’ post-COVID recovery (Attenborough 2020; Georgieva 2020; Gates 2021), which governments have incorporated into policy statements of intent (HMT 2021; White House 2021a). Simply put, an economy based on green ideologies could avert climate and ecological disaster (Forster et al. 2020).

Since the 1980s, social and economic inequality has increased in numerous countries across the world (Piketty 2003; Atkinson et al. 2011; Parker 2014). Over the past 4 decades, the richest one percent have seen their share of national income increase by almost 300% (Joyce and Xu 2019). In contrast, lower waged workers are earning little more than their equivalents did in the 1990s (Joyce and Xu 2019), and it is these workers who have lost jobs as a result of COVID-19 disruption and should be at the forefront of any socially just Green Recovery plans.

Governments have pledged to build a green recovery from COVID-19 (HMT 2021; White House 2021b). However, details of what should constitute a green recovery are often vague, and the holistic environmental benefits of the approaches are unquantified. For example, while the energy output of a windfarm can be accurately estimated, the environmental effects of resource use in manufacturing, consumer behaviour in relation to changes in employment and other factors become increasing hard to estimate. The lack of holistic understanding of these actions has resulted in different environmental ideologies, from those dedicated to decoupling carbon output from economic growth through to those calling for a degrowth strategy (e.g. Fischer-Kowalski et al. 2011; Sandberg et al. 2019). Our recent research (conducted just prior to COVID) has provided an initial ‘semi-quantification’ of different environmental approaches and has demonstrated that comprehensive Green New Deal (GND) strategies, focussing not only on renewable energy and insulation, but also on carbon taxation, and ending fossil fuel subsidies, combined with comprehensive Nature-based solutions are likely the best outcome for the environment (Stafford et al. 2020). These solutions also have social benefits by reducing social inequality and creating ‘communities’, for example, by enhancing local food production. However, they are likely to result in static levels, or slight decreases, in GDP, rather than following the current paradigm of economic growth. Here, we extend this research to investigate which jobs and industries can best be included in green recovery plans, especially those of Global North countries, to promote working, reduce social inequality and ensure environmental benefits.

Different countries and different NGOs and think tanks provide a wide range of strategies, and it is not possible here to evaluate all of these. However, we evaluate a range of options, largely based on increasing complexity of environmental interventions, based on approaches listed in government policy, and from a wide variety of NGOs promoting environmental ideas. Renewable energy is often the cornerstone of government net-zero approaches and is presented as a way of reducing carbon emissions as well as creating jobs and economic growth (e.g. HMT 2021) and forms the first section of our analysis. However, typical GND strategies, such as that presented to the US House of Representatives, also consider energy efficiency, through better insulation of homes and workspaces, use of heat-pump technology for heating, better use of raw materials through better waste management (i.e. increasing the circular economy) and investment in public transport (e.g. Congress Bill H.Res.109 2019). This GND strategy, with investment in more areas, alongside jobs in these areas forms our second scenario. The ‘comprehensive’ GND, including large carbon taxes and ending fossil fuel subsidies as promoted by several NGOs (e.g. Green New Deal for Europe 2019) forms our third scenario.

Nature-based solutions have received considerable interest as a climate mitigation tool (e.g. Stafford et al. 2021; House of Lords 2022), but have also been shown to work through different mechanisms to other GND strategies (Stafford et al. 2020). Research has demonstrated how cost-effective investing in nature conservation jobs can be, especially in areas where both biodiversity and carbon sequestration can be enhanced (Dicks et al. 2020; Clavey et al. 2021), yet specific investment in these jobs is often lacking. Given the importance of nature-based solutions, we examine the role of investment in these, alongside conservation jobs as our fourth scenario, and the inclusion of these investments alongside the previous comprehensive GND strategies as our fifth scenario. For our sixth scenario, we also examine the impact of low carbon jobs, not necessarily associated with carbon reduction (e.g. social care or teaching), such an approach has been promoted under a ‘Green Jobs For All’ slogan in the UK by the Green New Deal UK group (Green New Deal UK 2020), and is considered a method of increasing employment without damaging the environment.

Finally, scenarios seven and eight compare the possible ‘green recovery’ scenarios to more typical recovery scenarios, such as increasing manufacturing directly (not just in green industries) or economic strategies to boost economic growth.

## Methods

**Fig. 1:**
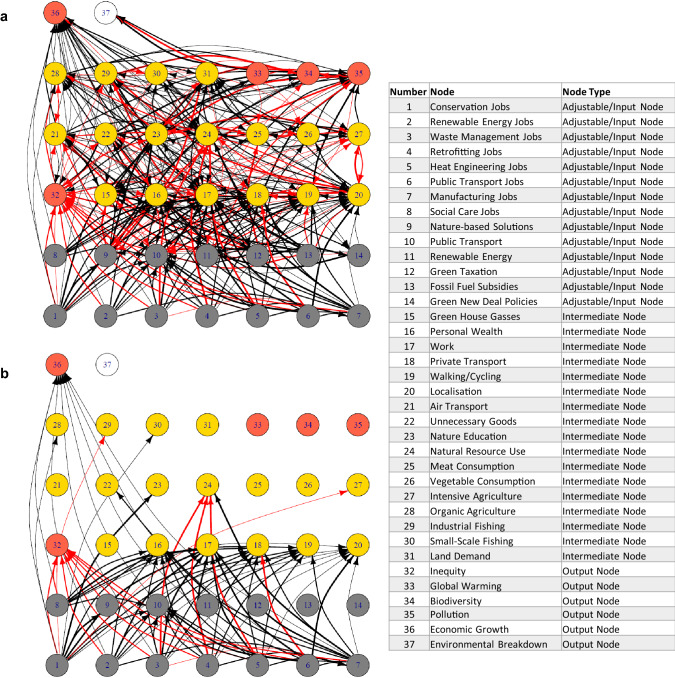
Visualisation of the Bayesian belief network model. Circles represent nodes, with numbers and node types referring to the variables in the legend (Grey = adjustable/input node, yellow = intermediate node, orange = output node, white = overall risk of environmental breakdown—also an output node). Red arrows represent negative interactions between nodes and black arrows positive interactions. Line thickness indicates the strength of interaction (slight, moderate and strong). **a** Full network model. **b** additional modifications from model presented in Stafford et al. (2020), nodes 1–7 representing job types are new

In this study, we have expanded our existing models, based on Bayesian belief networks (fully described in Stafford et al. 2020), which consist of a series of ‘nodes’ connected by weighted ‘edges’ (circles and connecting lines, respectively, in Fig. 1). The weights of the edges are based on changes likely to occur to ‘child’ or receiving nodes, given a change in the ‘parent’ or originating node, are either strong, medium or weak in value, and are either positive interactions (when the parent node increases, it is most likely that the child node also increases) or negative interactions (when parent node increases, the child node will likely decrease). Some nodes are given ‘prior’ values and these values progress through the network, with child nodes becoming parent nodes for subsequent interactions. The priors changed depend on the scenarios investigated and are given in Table 1. The full mathematics of Bayesian belief networks used in this study are given in Stafford et al. (2020), and a working model based on a Microsoft Excel template, and indicating all edge interaction strengths can be found in the Supplementary Material.

**Table 1:** Scenarios implemented in the Bayesian belief network

Scenario	Specific changes to model priors
1. Increase renewable energy and renewables jobs	Node 2 = 0.9 Node 11 = 0.9
2. Increase Core GND and GND jobs	Node 2 = 0.9
These encompass: renewable energy jobs	Node 3 = 0.9
Waste management jobs	Node 4 = 0.9
Retrofitting jobs	Node 5 = 0.9
Heat engineering jobs	Node 6 = 0.9
Public transport jobs	Node 14 = 0.9
3. As scenario 3 but also increasing green tax and reduction of fossil fuel subsidies	Node 2 = 0.9
	Node 3 = 0.9
	Node 4 = 0.9
	Node 5 = 0.9
	Node 6 = 0.9
	Node 12 = 0.9
	Node 13 = 0.1
	Node 14 = 0.9
4. Increase nature-based solutions and conservation jobs	Node 1 = 0.9
	Node 9 = 0.9
5. As scenario 4 but adding nature-based solutions and conservation jobs	Node 1 = 0.9
	Node 2 = 0.9
	Node 3 = 0.9
	Node 4 = 0.9
	Node 5 = 0.9
	Node 6 = 0.9
	Node 9 = 0.9
	Node 12 = 0.9
	Node 13 = 0.1
	Node 14 = 0.9
6. As scenario 5 but also including social care jobs	Node 1 = 0.9
	Node 2 = 0.9
	Node 3 = 0.9
	Node 4 = 0.9
	Node 5 = 0.9
	Node 6 = 0.9
	Node 8 = 0.9
	Node 9 = 0.9
	Node 12 = 0.9
	Node 13 = 0.1
	Node 14 = 0.9
7. Increasing manufacturing jobs only	Node 7 = 0.9
8. Increasing economic growth	Node 36 = 0.9

Description of the type of financial stimulus and how this was incorporated by altering network nodes

*Node* numbers refer to those indicated in Fig. 1

The models can be thought to provide a convenient way of analysing complex systems, such as an environmental-socio-economic model, with limited data and utilising expert opinion where necessary. While results are not fully quantitative, they can be considered ‘ordinal’, so ranking of different scenarios, for example, with respect to outcomes for climate change, biodiversity, pollution, social inequality, or levels of economic growth can be easily conducted. We specifically mapped the creation of jobs in nature conservation, renewable energy, waste management, retrofitting of building insulation, heat-pump installation, public transport, manufacturing and social care into our previous model, acknowledging that some of these green jobs would have indirect effects on manufacturing (e.g. to produce heat pumps) (Fig. 1).

We analysed different scenarios, such as investment in conservation jobs and nature-based solutions only, investment in renewable energy and renewable energy jobs only, investment in ‘core’ GND scenarios (all jobs listed above, except nature conservation, social care and [directly] in manufacturing), investment directly in manufacturing in general and combining GND jobs with nature conservation and social care (Table 1). Based on our previous work, which demonstrated GND strategies were shown to only be effective when combined with economic change, such as high carbon taxes and ending fossil fuel subsidies, we also investigated the investment in jobs, alongside these economic measures (Table 1).

All prior nodes in the model were set to 0.5 (equal chance of increasing or decreasing) other than those described in Table 1. In a scenario where a node was likely to increase strongly (e.g. creation of renewable energy jobs in scenario 1), it was given a value of 0.9 (strongly increasing). Where a node was likely to decrease significantly (e.g. reduction of fossil fuel subsidies in scenario 3), it was given a value of 0.1 (strongly decreasing). Only the nodes indicated in Table 1 were adjusted as priors in the model.

## Results

**Fig. 2:**
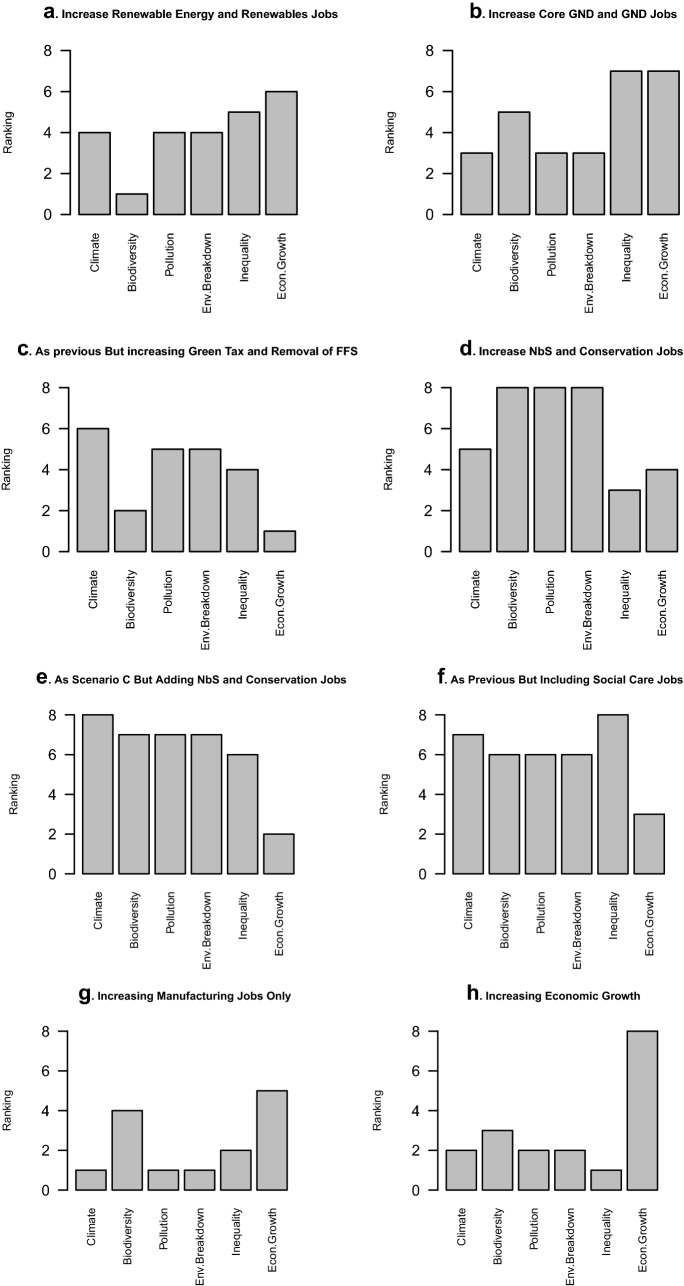
Predicted outcomes of different simulations for COVID recovery for selected variables. Y axis represents relative scoring of variable across all eight simulations where score of 8 is highest. High values represent ideal scenarios for variables (e.g. high scores indicate reduction of the risk of climate change, increases in biodiversity, reduction of social inequity)

Increases in renewable energy and renewable energy jobs resulted in increased economic growth and reduction in social inequality (Fig. 2a). As more GND job areas were added, economic growth increased further, and levels of social inequality fell. However, environmental benefits ranked poorly (Fig. 2b). The inclusion of ending fossil fuel subsidies and high carbon taxes in our models curbed economic growth and increased the environmental benefits (Fig. 2c). Increases in nature conservation jobs and nature-based solutions produced very good overall environmental outcomes (although only the third best for climate, but high for biodiversity gains and reduction of pollution). However, this approach, considering a limited number of jobs in just one sector, did not contribute greatly to a green recovery, with poor scores for decreasing social inequality (Fig. 2d). The strongest environmental benefits overall, including the highest score for climate and second highest scores for other environmental issues, were obtained by investment in the full suite of GND jobs and GND areas, alongside conservation jobs and nature-based solutions, combined with carbon taxes and ending fossil fuel subsidies (Fig. 2e). However, only small reductions in environmental benefits, alongside further reduction in social inequality, were seen with the additional inclusion of social care jobs (Fig. 2f). While manufacturing jobs indirectly increased as a result of investment in other areas, a direct targeted investment in manufacturing (rather than as a result of the need to develop environmental products such as renewables and heat pumps) resulted in a poor outcome for the environment (Fig. 2g). Similarly, a strategy to pursue economic growth through any means, rather than through green growth, also resulted in poor environmental outcomes (Fig. 2h).

## Discussion

The results demonstrate that investment in nature and in the principles of a comprehensive GND (including carbon taxation and ending fossil fuel subsidies) are essential for the future of the environment. Green economic recovery can be achieved through a direct investment in jobs in these areas, and the economic benefits of green recovery can be increased further through investment in other low carbon jobs, such as social care, with only minor environmental trade-offs. However, in this study, we consider green recovery economic success as a reduction in social inequality (due to the need to create jobs for lower paid workers to counter those lost through COVID). Economic growth (i.e. increases in GDP) tends to reduce any environmental gains found from green technology, circular economy approaches and GND investment (due to greater use of resources and demand in the economy overall), however, increases in carbon taxation and ending of fossil fuel subsidies can curb economic growth, increase environmental benefits and provide a mechanism to finance the investment in conservation and GND processes and jobs.

Along with previous studies (e.g. Stafford et al. 2020), these results provide some of the first (semi-) quantitative approaches to holistically examining the effectiveness of environmental policies, and the first to examine green recovery schemes in this manner. While there have been economic analyses of these schemes (e.g. Cambridge Econometrics 2020; Pollitt et al. 2021), the environmental benefits are often focussed only on carbon emission reductions, rather than the environment more holistically (for example, Politt et al. 2021 refer to car scrappage schemes and promotion of electric vehicles, despite issues with pollution, biodiversity loss and resource use associated with their manufacture). Furthermore, while studies such as Politt et al. (2021) do not investigate a full range of GND policies and associated employment, the results for less comprehensive GNDs (e.g. focusing on renewable energy) are consistent with the results from this study, both showing immediate increases in jobs and GDP, and at a modest reduction in CO_2_, but better environmental outcomes than other economic growth scenarios.

The need for rapid and strong focus on climate mitigation, pollution reduction and curbs on biodiversity loss are scientifically well established (e.g. IPBES 2019; IPCC 2022). However, despite Global North government pledges, COVID recovery so far has not focussed on these ‘green’ sectors. Approximately, 30% of the $14.9 trillion total COVID stimulus packages have gone to agriculture, energy, industry, transport and waste—all of which have a profound impact on nature and greenhouse gas (GHG) emissions. Overall, the net impact to the environment of this recovery will be negative, and just 10% of the global stimulus is directed to restoring nature and cutting GHG emissions (VividEconomics 2021). While the pandemic initially cut global carbon emissions in Q1 and Q2 of 2020, these levels rebounded quickly, with global figures higher than in 2019 by Q3 of 2020, and China’s emissions increasing rapidly in Q2 (due to early strict lockdowns in Q1, IEA 2020).

Without intervention, and strong policies to ‘build back greener’ traditional economics applied to fossil fuels will result in rapid rises in carbon emissions post any economic slowdown. Both COVID and the financial crash of 2008 resulted in big (> 30%) reductions in the price of crude oil (Li & Li 2021). The economic laws of supply and demand, therefore, mean any recovery will naturally take full advantage of these cheaper ‘dirty’ energy costs and result in increased carbon production until prices stabilise. However, due to a variety of global supply issues, current prices of gas and oil at record levels (as of August 2022), there has never been a more opportune time to enact a just transition towards a society built on green fundamentals.

While the chance to prevent an immediate recovery of carbon emissions post COVID (for example, by applying higher carbon taxes) has passed, it is now that many countries are seeing economic problems such as high cost of living increases, supply chain issues and high levels of inflation (Roy-Mukherjee 2022). Within the UK, for example, there is also a strong narrative for ‘levelling-up’ of different parts of the country, where some traditional industries have been badly affected, partly through COVID, but also through gradual decline over many years (DLUHC 2022). An approach such as ‘levelling up’ gives a real opportunity to introduce green industries, such as construction of renewable energy products, while providing much needed jobs, although within the UK the plans have fallen short of these targets (Ainscough 2022).

‘Green’ measures have traditionally focused on cutting GHG emissions, compared with improving biodiversity and nature. However, in this study, we show the important role of investing in nature-based solutions and conservation in tackling the entire environmental crisis. Just 20% of the green stimulus to date (US$667 billion) has been spent on protecting ecosystems and increasing biodiversity. This amount ($141 billion) is only 55% of the amount than has been spent on measures which will increase habitat loss and pollution ($262 billion), both of which will degrade natural capital and reduce biodiversity. In addition, only 30% of nations have invested so far in establishing and protecting effective nature-based solutions (Vivid Economics 2021), despite clear evidence of their cost effectiveness in combatting biodiversity loss and climate change (Clavey et al. 2021).

An important outcome of this study is the need to curb economic growth to achieve the biggest benefits of additional climate action. Our economic growth node is not directly connected to any of the environmental output nodes in the model, and increases in economic growth are associated with some positive environmental outcomes such as greater green taxation and more investment in renewable energy (as per Panayotou 2000; Everett et al. 2010). However, it is likely the connection between economic growth and greater natural resource use which results in poorer environmental outcomes (Everett et al. 2010; Hickel 2018). Currently, evidence for ‘decoupling’ of economic growth from environmental degradation is extremely weak (Parrique et al. 2019; Sandberg et al. 2019), and without new and compelling evidence to support this, then it must be assumed that ‘degrowth’ strategies (i.e. economic strategies which do not actively focus on economic growth, Raworth 2017; Hickel 2018) must become incorporated in economic thinking if we are to avoid environmental breakdown.

While this study has focussed on Global North economies, environmental and socio-economic crises are prevalent globally. Investment in green industry is vital, however, in the Global South, the need for economic growth may be higher than in Global North countries (Roy 2016), and these countries already have significantly lower per capita carbon footprints. It is, therefore, worth noting that boosting the economy through green jobs, without the inclusion of measures such as taxation to curb economic growth, produces better environmental outcomes (alongside the second highest level of economic growth), compared to measures such as investment in manufacturing jobs outside of green industries. While care that ‘green technology’ does not result in exploitation of the Global South for resources, nor create an environmental disaster due to pollution from issues such as poor mining practices, this illustrates how countries can develop economically while minimising their environmental impact.

In summary, while earlier action on post-COVID recovery of carbon emissions would have been useful in addressing climate targets, there is still much to be gained by including a strong green recovery at the international level to a world beginning to emerge from the COVID-19 pandemic, and a world affected by the supply of fossil fuels. These benefits apply both to the environment, and to society, but need strong political action to provide meaningful environmental gains. Investment in green jobs, which must include nature, is key, but so is economic reform, and the need to increase carbon taxation, end fossil fuel subsidies, and find different measures of societal progress, beyond the economic growth paradigm.

## Supplementary Information

Below is the link to the electronic supplementary material.

Supplementary material S1. Excel workbook (note contains macros) of the modified Bayesian belief network model. Input parameters (levels tab) have been set to values in Table 1 to perform scenario evaluations with other input parameters set to 0.5. Edge strengths and directions can be found in the Interaction Probabilities tab (+ ve strong = 0.85, + ve medium = 0.75, + ve weak = 0.65, -ve strong = 0.15, -ve medium = 0.25, -ve weak = 0.35) (XLSM 88 KB)

## Data Availability

All data generated or analysed during this study are included in this published article and its supplementary information files.
